# Supplementary material for: Chromothripsis during telomere crisis is independent of NHEJ, and consistent with a replicative origin
Source: Genome Res. 2019 May;29(5):737–49. doi: 10.1101/gr.240705.118 (PMC6499312; doi:10.1101/gr.240705.118)
Supplement: Supplemental Material [file supp_gr.240705.118_Supplemental_file_1.zip › contigs/annotated_contigs/DB116/contig.2.DB116_length_541_mean_cov_8.40295748614.docx]

**DB116_length_541_mean_cov_8.40295748614**

```
CCGGCTAATTTTTTGTATTTTTAGTAGAGACGGGGTTTCACCTTGTTAGCCAGGATGGTCTCGATCTCCTGACCTCATGATCCACCCGC
 >chr10:4572571-4572714 - E=2e-74
CTCGGCCTCCCAAAGTGCTGGGATTACAGGCGTGAGCCACCGCGCCCGGC|CATG|AAATTTTAAAGTCTAAATTATATTGATTAAGAA
                                                   >chr1:100993819-100994221 - E=2e-230
ACAAGAAAACCAGGAGCTACCACCCTGTTTCTACGTCACTGAGGTCATCATTGATGGTGAAAAAAAATTACTTCTATTTGCTGTATCTC

AGAAATAGCCTGTCTTCTCCCTGCTTCACAGCAAATTGGCACTCTCAATTTCCAGAAAACCCTTTCTCAGCTATAGTCCTTGAAAATTT

TGAAATCCTTCTCTGGATACTGGGCACAACCTTCTGAGCCTCAGTGAAAGCAATGGCCAAATATGTGTTAGAAGCCTCTGCTGTGGAAT

TTCAGAGGTACAGTCATCACCCCATTTCTGCTGCTTATCAGACATCTCTTAGATATCCCTCAAACAGCATAAACCAAACTAAACTCCCT

GTGCTTGCA
```
